# Insecticidal Action of Local Isolates of Entomopathogenic Fungi Against *Bactrocera oleae* Pupae

**DOI:** 10.3390/biology14010005

**Published:** 2024-12-24

**Authors:** Spiridon Mantzoukas, Alexandros Margaritis, Thomais Sourouni, Vasiliki Georgopoulou, Chrysanthi Zarmakoupi, Vasileios Papantzikos, Ioannis Lagogiannis, Panagiotis A. Eliopoulos, George Patakioutas

**Affiliations:** 1 Department of Agriculture, University of Ioannina, Arta Campus, 47100 Arta, Greece; agu16208@uoi.gr (A.M.); agu16281@uoi.gr (T.S.); agu16148@uoi.gr (V.G.); chris.zarm@hotmail.com (C.Z.); b.papantzikos@uoi.gr (V.P.); gpatakiu@uoi.gr (G.P.); 2 ELGO-Demeter, Plant Protection Division of Patras, NEO & L. Amerikis, 26444 Patras, Greece; lagoipp@gmail.com; 3 Laboratory of Plant Health Management, Department of Agrotechnology, University of Thessaly, Geopolis, 41500 Larissa, Greece; eliopoulos@uth.gr

**Keywords:** entomopathogenic fungi, *Bactrocera oleae*, pupae, soil

## Abstract

In this work, the ability of certain entomopathogenic fungi to control the olive fruit fly *Bactrocera oleae* (Diptera: Tephritidae), which significantly affects olive cultivation, was assessed. First, entomopathogenic fungi that often contribute to reducing pests, as well as *B. oleae*, were sought out. Puparia of *B. oleae* were collected from oil mills, and soil samples were collected from various olive grove regions of Greece. These soils were used as the substrate in which the effect of the entomopathogenic fungi on the *B. oleae* pupae was studied. In addition, the same treatments were studied in non-soil conditions. The results show that certain known entomopathogenic fungi are able, in their natural environment, to kill *B. oleae* pupae to a significant extent. The survival time for adults (days) was lower in the presence of *Beauveria bassiana* (Hypocreales: Cordycipitaceae), at 13.20 in the soil (5.56 ± 0.42) and non-soil (6.76 ± 0.46) conditions, compared to that of the control in soil (9.83 ± 083) and non-soil (9.90 ± 0.72) conditions. This is encouraging for Mediterranean regions where the presence of *B. oleae* significantly affects olive cultivation, while quality olive oil is in high demand.

## Simple Summary

Olive cultivation in Greece suffers every summer due to the olive fruit fly. The damage it causes is usually noticed about two months before harvest, when the pest’s population has increased considerably. There are various methods for limiting the olive fruit fly, but no one has been able to completely eliminate this problem to date. This insect pupates in the soil, which is the natural environment of entomopathogenic fungi. Therefore, it is an advantage for entomopathogenic fungi to be used to control the olive fruit fly at this early stage before it even proceeds to harm the olive fruits. In this study, we tried to simulate the real conditions in which the pest pupates and the entomopathogenic fungi inhabit using soil samples. They could be used as an alternative to pesticides, which, due to their overuse, alter olive oil quality.

## 1. Introduction

Olive farmers must deal with obstacles that reduce olive oil production. A crucial cause of damage is the olive fruit fly *B. oleae*. This insect causes intense impairment at all stages, reducing the annual olive production by up to 15% in the Mediterranean region [1]. *B. oleae* is a monophagous pest of olive plants that causes damage by chewing the pulp of the fruit and harming it, while females lay their eggs right beneath the olive fruit’s epicarp [2], leading to fruit infestation. *B. oleae* pupates over winter. The first generation comes in spring, and during the summer, two to five generations may persist (varying according to the climate conditions), which damage the olive fruits during their pit-hardening stage. In this period, adults oviposit eggs inside the olive fruits. As autumn comes, and fruit-ripening follows, the level of damage peaks, and then damage stops when new nymphs come out of the fruit to pupate in the soil [3]. Female *B. oleae* can oviposit 12 eggs a day and about 200–250 eggs in their lifetimes [4,5]. In order to feed their larvae after hatching, they create tunnels throughout the fruit’s mesocarp, consuming the olive fruit pulp, and this deterioration makes olive fruit susceptible to subsequent infestations by phytopathogenic fungi [6]. At the third larval instar, they exit the fruit and pupate on the ground [7,8,9]. Qualitative and quantitative injuries during fruiting are inevitable as larval growth stages progress in the fruit tissues, inducing severe damage, altering the expression during biosynthesis of volatile and phenolic compounds, and altering the olive oil composition [10], which usually results in reduced oil production [11]. It is estimated that *B. oleae* has caused serious economic damage in the Mediterranean basin, costing more than USD 1 billion per year [1].

The use of organophosphate insecticides and pyrethroids to manage *B. oleae* in Greece has become an issue, on the one hand because this treatment is not pest-targeted [12] and on the other hand because over the long-term use of these chemicals, their efficiency against the *B. oleae* population gradually decreases [13,14]. This concern has led the olive-growing industry to rationalize the overuse of pesticides, aiming towards integrated pest management (IPM) principles [15,16,17,18]. Pyrethroid insecticide resistance has evolved in *B. oleae* populations in Greece, threatening the efficacy of control interventions based on this class of insecticides [19]. In recent years, Kampouraki et al., 2018, in a nine-year-long survey of the olive fruit fly, observed an increase in its pyrethroid resistance levels over time [13]. Crucial levels of resistance to pyrethroids have previously been reported in *B. oleae* populations from Greece [20,21].

In the face of this upcoming change in olive culture management, entomopathogenic fungi (EPFs) seem an eligible candidate for experimentation as an alternative for biological control of *B. oleae* [22]. EPFs act through the pest’s cuticle, working effectively as bioinsecticidal agents and controlling the soil-dwelling stages of insects [23]. There are a plethora of findings supporting the efficiency of EPF strains against Tephritidae and *Bactrocera* sp. during the pupal stages [24,25,26]. *Metarhizium anisopliae* (Hypocreales: Clavicipitaceae) and *B. bassiana* in particular have had their efficacy against Tephritidae puparia confirmed [27,28]. Many EPFs have been mentioned due to their contact efficacy against *B. oleae* pupae such as *Beauveria brongniartii* (Hypocreales: Cordycipitaceae) and *B. bassiana,* as well as additional fungal species such as *Mucor hiemalis* (Mucorales: Mucoraceae), *Penicillium aurantiogriseum*, and *Penicillium chrysogenum* (Eurotialles: Aspergillaceae) [29]. Other biological control methods for *B. oleae* are the use of *Bacillus subtilis* (Caryophanales: Bacillaceae) at the larval stage [30] and *Bacillus thurigiensis* (Caryophanales: Bacillaceae) in field spraying applications [16]. Moreover, efforts have been made to reduce the *B. oleae* population using the parasitoid *Psyttalia ponerophaga* (Hymenoptera: Braconidae) [31].

Furthermore, it has been suggested that control of *B. oleae* puparia through the soil inoculation of EPFs may be more effective because they may survive longer in the soil when recycled in insects or roots [32]. The soil environment preserves EPFs under the ideal temperature and moisture conditions and further protects their survival by shielding them from UV radiation [33]; thus, inoculation of the substrate is an ideal medium for EPF. The level of mortality of the oriental fruit fly, *Bactrocera dorsalis* (Diptera: Tephritidae), during pupation in insect-pest-suppressive soils [34] inoculated with *B. bassiana*, *Metarhizium robertsii* (Hypocreales: Clavicipitaceae), *Isaria fumosorosea* (Hypocreales: Cordycipitaceae), and *Glomus* spp. (Glomerales: Glomeraceae) indicated success. *B. bassiana* WG-18 and WG-21 have been shown to be lethal to *Bactrocera zonata* (Diptera: Tephritidae) and *B. dorsalis* [35,36]. In the same pests at the pupal stage, successful efficacy in terms of the mortality of *B. bassiana* WG-18 and *M. anisopliae* WG-02 when they were combined with nematodes was confirmed in the study by Wakil et al., 2022 [37]. The extensive use of aerial chemical insecticide spraying for more than 60 years is a reason for the aggravation of the *B. oleae* population [38]. Most chemical sprays only target adults, while after the autumn, the last generation’s larvae fall to the soil to pupate and survive there; thus, the soil is the best substrate in which to control *B. oleae* [39,40]. In the work by Yousef et al., 2013 [38], the use of the EPF *Metarhizium brunneum* (Hypocreales: Clavicipitaceae) successfully reduced the adult *B. oleae* spring population by 50–70%, showing this to be an effective, economically viable, and environmentally friendly method for *B. oleae* management.

Finally, the number of active ingredients approved in the EU for pest management has been reduced. There are only 500 substances available [41], and among these, some are microorganisms. That is why we believe that research on EPFs as viable alternatives for pest management is more important now than ever. Nevertheless, there is a lack of studies regarding the control of *B. oleae* puparia using soil-inoculated EPFs, and this is a research object that may offer adequate strategies for managing a preeminent pest of olive fruits with the intention of improving olive production. This could be highly beneficial, particularly for Mediterranean countries, where the demand for olive oil is increased. The purpose of this study was to investigate the effect of certain EPFs on *B. oleae* puparia in soil conditions.

## 2. Materials and Methods

### 2.1. Rearing of Bactrocera Oleae 

Olive fly pupae were collected in November 2021 from oil mills in the Preveza region (Greece) and routinely transferred to the laboratory within 24 h. Then, to obtain same-aged cohorts, emerged flies were reared in 30 × 30 × 30 cm^3^ net cages in a growth chamber (PHC Europe/Sanyo/Panasonic Biomedical MLR-352-PE) in controlled environmental conditions, at 23 ± 2 °C and 65 ± 10% RH and with a 16:8 (L:D) h photoperiod. Female and male flies were reared in the same cage, and they were fed on a dry diet consisting of sugar and yeast extract (Sigma-Aldrich, Burlington, MA, USA) (4:1). Water was constantly available on a sponge wick and refreshed every 7 days. To obtain individuals of the same age, sand from the rearing cages was sieved three times a day, and the young pupae collected (age < 8 h) were maintained in small Petri dishes. They were used for experimentation after 4–5 days.

### 2.2. EPF Cultures

We obtained sixteen strains of EPFs, belonging to the genera *Beauveria*, *Cordyceps*, and *Metarhizium*, from the personal collection of the first author (Table 1).

### 2.3. Lab Culture Fungal Isolates

SDA was used as the medium for the cultivation of the EPF isolates in 9 cm Petri dishes for 15 days at 25 °C and 65°H relative humidity. To prevent contamination, the Petri dishes were sealed with Parafilm^®^ (American National Can, Chicago, IL, USA). The isolates were morphologically identified using a ZEISS Primo Star microscope (Carl Zeiss Microscopy GmbH, Oberkochen, Germany) at 400× magnification after they were subcultured numerous times on plates with SDA to assure their purity, and genomic DNA (gDNA) was isolated from the monosporic cultures [42,43]. Fresh conidia were harvested after 15 days by scraping the Petri dishes’ surfaces with a sterile loop and placed in a 500 mL glass beaker containing 50 mL of sterile distilled water and 0.05% Tergitol^®^ NP9 (Sigma-Aldrich, St. Louis, MO, USA). A magnetic stirrer was used to mix the conidial suspension for five minutes after filtering it through sterile cloth layers. A Neubauer hemocytometer was used for the measurement of the fungus conidia concentration (a WEBER SCIENTIFIC hemocytometer for cell counting, Hamilton Township, NJ, USA). Dilution was carried out by adding 10 mL of the conidial suspension to the required amount of sterile water, resulting in a final concentration of 10^8^ conidia per ml for the fungal isolates. This specific concentration was chosen due to its widespread use in numerous relevant studies, and the conidial viability exceeded 97% for all of the fungal isolates.

The viability of all of the fungi tested was determined by spreading a 100 μL aliquot of the conidial suspension (1 × 10^6^ conidia mL^−1^), prepared with a sterile surfactant solution (0.1% *v*/*v*) of Tween 80, onto SDA medium in a Petri dish (90 × 15 mm) and incubating it in the dark at 25 ± 1 °C. The SDA plates of the tested fungi were incubated for 18 h prior to their evaluation. Conidia were scored as viable if any germ tube was 2× longer than the diameter of the spore; a total of 100 conidia was evaluated per sample under 400 × magnification. Conidial viability was calculated based on the formula below:

Viability (%) = [G1/(G1 + G2)] × 100

where G1 refers to the number of germinated conidia, and G2 is the number of non-germinated conidia, while the sum of G1 and G2 is equal to 100. Thus, the percentage of viable conidia was determined by counting a total of 100 conidia per fungal sample. Fungal strains with ≥ 95% viability were used in the insect bioassays.

### 2.4. The Anti-Pupa Bioassay

The effectiveness of EPFs, as has been shown by many studies, does not depend as much on the dose as it does for chemical insecticides. We used one of the most common commercial dosages so that our results would have practical relevance. A dose–response study would have required a vast number of measurements, and it is highly doubtful whether it would have offered any useful results. Sixteen EPF isolates were evaluated in terms of their efficacy against the pupae of *B. oleae*. The collection of the pupae was simple, and there was no need to handle them manually: puparia were collected from the lab rearing environment using a fine brush wetted with distilled water. The bioassay environment involved 4–5-day-old pupae, which were buried in sterilized soil from olive cultivation at a depth of 3 cm for the first treatment, while the second treatment took place without soil. Briefly, 2 mL of the conidial suspension (1 × 10^8^ conidia mL^−1^) was sprayed using a Potter spray tower (Burkard Manufacturing Co. Ltd., Rickmansworth, Hertfordshire, UK) at 1 kgF cm^−2^ onto the pupae on the surface with soil and the surface without soil. After mixing, the pupae (4–5 days old) were buried individually in cups at a 3 cm depth, and the cups were covered with lids. The control group was sprayed with aqueous solution with 0.05% Tergitol^®^ NP9 (Sigma-Aldrich, St. Louis, MO, USA) onto the soil surface and the pupae directly. Pupae that were unable to emerge as adult flies were considered dead. Upon their emergence, adults were transferred into cages (30 cm × 30 cm × 30 cm) and provided with water and adult food, and their mortality was recorded over 10 days. Adult mortality and mycosis were determined daily, and all dead individuals were removed from the cages each day. Individuals from each developmental stage (adult or pupal) were placed inside plastic Petri dishes lined with sterile and moist filter paper (Whatman^®^ Sigma-Aldrich, St. Louis, MO, USA). These dishes were wrapped with Parafilm and then incubated at 25 °C to observe the presence of fungal outgrowth. Before putting them into the plastic Petri dishes, the pupae and adults were surface-sterilized with 1% sodium hypochlorite, followed by three rinses with distilled water. Twenty individuals were used for each treatment replicate. There were ten replicates for each treatment, and the whole experiment was conducted twice, resulting in twenty replications (200 individuals were used for each treatment). Pupa hatch time, the presence of mycelium on dead pupae, the duration from pupation to adult emergence, adult survival time, and the presence of mycelium on dead adults were determined.

### 2.5. The Statistical Analysis

All values were arcsine-transformed prior to the analysis. The data were analyzed using a two-way ANOVA using the general linear model in SPSS (SPSS Inc., Chicago, IL, USA, version 24) (IBM, 2019). In the case of significant F values, means were compared using the Bonferroni test. The Kaplan–Meier method (for life parameters) was also selected to determine the median lethal time for *B. oleae* following the application of the pathogen concentrations. The Cox regression [44] method was selected to determine the hazard effect of the isolates on *B. oleae*. This is a survival analysis regression model that describes the relation between the incidence of events, as expressed by a hazard function and a set of covariates. A comparison of the survival distributions was made using the Breslow (generalized Wilcoxon) test (SPSS v.23.0).

Survival data are generally described and modeled in terms of two related probabilities: survival and hazards. The survival probability (which is also called “the survivor function”), S(t), is the probability that an individual survives from the origin time (e.g., the beginning of a treatment) to a specified future time, t.

The hazard probability is usually denoted as h(t) or λ(t) and refers to the probability that an individual who is under observation at time t experiences a given event at that time. It represents the instantaneous event rate for an individual who has already survived by time t. Τhus, while the survivor function reflects the cumulative non-occurrence of an event, the hazard function focuses on the occurrence of an event. The mathematical expression of the Cox model is as follows:

h(t) = h0(t) × exp{b1x1 + b2x2+⋯+bpxp}

where the hazard function h(t) is dependent on (or determined by) a set of p covariates (x1, x2, …, xp), whose impact is measured by the size of the respective coefficients (b1, b2, …, bp). The term h0 is called the baseline hazard and is the value of the hazard if all xi values are equal to zero (the quantity exp (0) equals 1). The “t” in h(t) reminds us that the hazard may (and probably will) vary over time.

## 3. Results

**Figure 1:**
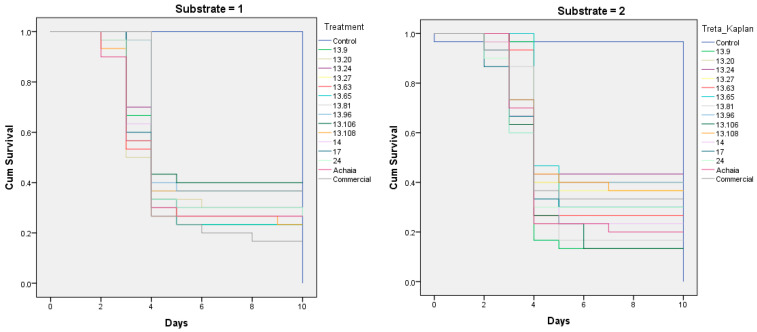
The survival of adults of *B. oleae* in two different substrates was monitored for 10 days.

The virulence of several isolates of *B. bassiana*, *B. varroae*, *M. anisopilae*, *M. robertsii*, and *C. blackwelliae* against *B. oleae* pupae in two substrates was estimated (Table 2). It indicated that the various fungal isolates affected the survival time of the adults in diverse ways (Figure 1).

Accordingly, in terms of the highest pupa hatch time, this was estimated at 6.63 ± 0.48 days for *B. bassiana* 13.81 (in the soil) and 6.63 ± 0.41 days for *B. bassiana* 13.106 (in non-soil conditions). In all of the other isolates, the median lethal time exceeded 5 days (Table 3). The mycelial and conidial growth on the cadavers suggested that almost all of the deaths were pathogen-related.

Observations of the pupae showed that external mycelium appeared within the first 72 h after placing them on the moist filter paper. The results for the soil substrate showed that external fungal growth was observed in the treatments with *B. bassiana* 13.81 (100%) and 13.96 (90%), while the external fungal growth was not developed enough in the *B. bassiana* Achaia (40%)-treated pupae. The average percentage of mycelial growth on the dead pupae treated with the other isolates ranged from 54.55% to 88.89% in the soil substrate. On the other hand, in the non-soil treatments, external fungal growth was observed for *B. bassiana* 13.20, 13.24, 13.106, and 13.108 (100%) and *M. robertsii* 24 (100%), while limited fungal growth was observed in the case of the *M. anisopliae* Crete (16.67%)-treated pupae. The mean percentage of mycelium growth on the dead pupae treated with the other isolates ranged from 37.50% to 90.91% in the non-soil substrate.

The highest adult survival time was estimated at 7.66 ± 0.43 days for *C. blackwelliae* (soil) and 7.433 ± 0.37 days for *B. bassiana* 13.27 (non-soil). In all of the other isolates, the median lethal time exceeded 5 days (Table 3). The mycelial and conidial growth on the cadavers suggested that almost all of the deaths were pathogen-related. Observations of the dead adults showed that external mycelium appeared within the first 96 h after placing them on the moist filter paper. The results for the soil substrate showed that external fungal growth was observed in the dead adults with *C. blackwelliae, M. robertsii,* and *M. anisopliae* (100%), while the external fungal growth was not developed enough with *B. bassiana* 13.9 (43.48%). The mean percentage of mycelium growth on the dead adults for the other isolates varied from 72.22% to 95.24% with soil as the substrate. On the other hand, external fungal growth was observed in the dead adults with *B. bassiana* strains 13.24 and 13.27 (100%) when using the non-soil substrate, while the growth was not sufficient for *B. varroae*, *M. robertsii*, *M. anisopliae*, and *B. bassiana* Achaia (0%). The mean percentage of mycelium growth on the dead adults for the other isolates varied from 10.53% to 71.43% with the non-soil substrate. The control hatch times were 2.87 ± 0.15 days (in the soil) and 2.60 ± 0.10 days (in non-soil). In the control treatment, no mycelium was found on the pupae or the dead adults.

The B coefficient for several isolates was positive, with Exp (B) > 1. Higher values are associated with an overall greater hazard and therefore a shorter survival for adult *B. oleae* and a longer hatch time for pupae. The *B. varroae* isolate represented a considerable hazard, with Exp (B) = 1.668. On the other hand, the control had a negative coefficient and the lowest Exp (B), and the survival was higher (Control Exp (B) = −2.479) (Table 4). Also, the key factors in the experiment were substrate, pupa hatch time, replication, adult survival time, the mycelium on the pupae, and the mycelium on the dead adults. The B coefficient for several factors was positive, with Exp(B) > 1. This state plays a crucial part in the effect of the pathogen. The replication factor was not important (Table 5).

## 4. Discussion

Our knowledge of the mechanisms controlling the interactions between insect pests and EPFs has greatly increased because of recent developments in the techniques for genetic, biochemical, physiological, behavioral, and ecological investigations [45,46]. In most of the cases studied so far, the infecting EPF changes its form from hyphal to unicellular after it penetrates the host’s exoskeleton. Inside the hemocoel, EPF cells move through the hemolymph, reaching and infecting the internal tissues [47]. This triggers several immunological responses in the insect host, which involve humoral and cellular mechanisms mediated mainly by hemocytes. In response to EPF infection, insects produce various components (lysozymes, transferrin, hemocyanin, etc.) via several pathways, like JAK/STAT, IMD, and others [48]. Nonetheless, EPFs have developed several defense mechanisms against insects’ immune responses, such as avoiding phagocytosis and encapsulation, hiding the EPF cells to evade detection, and generating toxins and other substances (proteins, secondary metabolites, laccase, PAMPs, enzymes, etc.) that inhibit the host’s responses [49,50]. Fungal growth can often overcome the host’s defense mechanisms through multiplication within the hemocoel. This infection stage is usually accompanied by changes in the insect’s behavior, like reproduction, response to chemicals, food consumption, development, etc. [46].

The highest toxicity (low survival time) of the EPFs used was found for pupa hatch time in *B. oleae*. The higher pupa hatch time and the greater presence of mycelium on these pupae confirm the findings of Bateman et al., 1996 [51], who found that the infection of insects by fungi depends on their biological stage. The findings of this study agree with those of various researchers [52,53,54] who found that *B. bassiana* and *M. anisopliae* conidia decrease the survival time of larvae and pupae of *Ceratitis capitata* (Diptera, Tephritidae). In general, the speed of mortality of the host is correlated positively with the conidial concentration [55]. In this work, survival time and mycelium presence, both in the pupae and adults, may be useful for determining specific clues of fungal–host interactions. The application of the conidial preparations to natural soil reduced the emergence of the insects and the adults’ life spans, representing a promising strategy for integrated management of the fruit fly. There is increasing evidence that habitat selection drives the pathogenicity of EPF species [56]. Thus, the results from our study indicate that screening of potential isolates should not be limited to those isolated from the original host.

To contribute to this goal further, the fungal inoculum may be disseminated by the adults before they are killed [57]. The highest toxicity (low survival time) of the EPFs used was found for adult survival time in *B. oleae*. Similar results were obtained by Konstantopoulou and Mazomenos 2005 [29], who, in evaluating the effect of different EPFs on adults of *B. oleae*, obtained lower survival times in the treated adults after 14 days of treatment. However, lower survival times were obtained using a walking bioassay on fungal colonies, where the time of exposure to the conidia was higher than that in the spraying bioassay. Equally, Siebeneicher et al., 1992 [58] reported that certain methods of applying conidial suspensions may be more effective than others because they cover more of an insect’s body area. The insecticidal efficacy of EPFs is highly influenced by several other factors, such as the insect’s behavior, population density, age, nutrition, and genetic information, as well as the effect of the host’s physiology and morphology on its sensitivity to biological control agents such as EPFs [59]. Therefore, differences in insects’ susceptibility to EPFs cannot be explained solely as a function of the conidial concentration applied [60].

Tephritids in the preimaginal stages, particularly puparia, are less susceptible to EPFs [39]. Our results disagree with those of Ekesi and Manianina 2007 [39]: we found that the puparia were affected by the presence of the EPFs. The high values of B and Exp(B) for each fungal isolate indicate high toxicity against the puparia of *B. oleae*. Significant variability in toxicity was detected among the various EPF isolates, with isolates 13.27, 13.108, 14, and 24 being more toxic against the puparia and emerged adults. According to Vanninen et al., 1999 [61], insects at the stages in which they live in the soil may have developed high levels of resistance to infection through natural selection because entomopathogens, especially fungi, are widespread in the soil. Furthermore, in tephritids, the cuticle of third-instar larvae remains to form the puparium, conferring a barrier to penetration and output of these fungal agents of microbial control. De la Rosa et al., 2002 [62] found that strains of *B. bassiana* effective against adults were not pathogenic against *Anastrepha ludens* (Diptera: Tephritidae) (Loew) puparia, while Cossentine et al., 2010 [63] also obtained strains of *B. bassiana* that were highly virulent against the adult western cherry fruit fly, *Rhagoletis indifferens* (Diptera: Tephritidae) (Curran), but that had low efficacy against its larvae and puparia. Investigations by Kaaya and Munyinyi, 1995 [64] in the tsetse fly (Diptera: Glossinidae; Glossina Wiedemann) also indicated the low susceptibility of its puparia to EPFs.

Sterilized soil was also used as the substrate to evaluate the effect of *M. brunneum* Petch on *B. oleae* (Rossi) in Córdoba, Spain [22]. The substrate treated with the EPF had a significant effect on *B. oleae*’s mortality since 82.27% of the treated individuals did not reach adulthood, while 64.55% reached this stage in the control treatment. In the Spanish study, the larval and pupal mortality in the control treatment was high (35.45%) compared to that in our study (12%). This makes the reduction in adult emergence of *Bactrocera carambolae* (Diptera: Tephritidae) by *M. anisopliae* (70%) in the present work greater in its absolute percentage compared to that obtained by these cited authors since they reached a reduction in emergence of 47%, while we found a decrease of 58%. The toxicity variables showed high values of B and Exp(B) for pupa hatch time, adult survival time, and substrate. These variables play a key role in the survival time of this insect. However, the fact that we used sterile soil in our work should be considered in future studies on controlling pupating larvae in a soil environment.

As expected, there was considerable variation among the isolates in their virulence against the *B. oleae* pupae. Such variations among various EPF strains of the same species against the same host have been well documented in many relevant assays. Many potential reasons for this variation have been suggested, with the most important being the original host of the EPF isolate, the size of the conidia, certain molecular and physiological mechanisms, germination speed, growth rate, and others [54,65].

Thus, it is important to emphasize the novelty of this study regarding the evaluation of the survival of adult *B. oleae* emerging from soil treated with EPFs since most previous studies have only considered the decrease in emerging adults without evaluating the subsequent adult mortality caused by the infection of the pupae still in the soil. In our study, the survival time of adult *B. oleae* in the sterile soil and non-soil treatments was low, between 5.3 and 7.9 days in the treated soil and non-soil conditions. On the other hand, for the control, the survival time of the adults was nearly 10 days. These results demonstrate the ability of the isolates to infect the larvae and pupae of *B. oleae* in soil and non-soil treatments under laboratory conditions. Another practical implication is the possibility of adults emerging from the soil treated with the EPF isolates used to actively transport the infective (conidia) parasitic EPFs to the pest’s concentration niches, causing healthy individuals to be infected (horizontal transfer), especially during copulation.

## 5. Conclusions

In conclusion, these findings marked a significant step in the development of ecologically friendly natural biocontrol EPFs for controlling the invasive fruit fly pest. It should be noted that our results are based on limited laboratory experiments under controlled conditions, which is not the case in nature. Apart from that, there are various restrictions on the utilization of EPFs as commercial bioinsecticides, such as their unstable effectiveness due to unfavorable environmental conditions, low killing speed compared with that of chemicals, the possibility for contamination with mycotoxins, etc. Our results suggest that we can consider the possibility of using biological control with EPFs as an alternative strategy for controlling *B. oleae* in Europe. Studies that examine these isolates under field conditions are needed to evaluate these fungi further as potential control agents for this fly. Based on the evidence, we may infer that EPFs are harmless and pose few hazards. Concerning the commercialization and registration of future isolates, the question is what further rules and testing are required to provide users and customers with a safe biocontrol product. This study will be helpful in the biological control approach of *B. oleae* and indicates the possibility of using these EPF isolates for biological control. We need to study and explore comprehensive methods of controlling this important pest in Greece and Europe. Within this spectrum, further trials should be conducted under various climatic conditions, testing additional fungal isolates or exploring combinations of EPFs with other biocontrol agents, which could enhance the effectiveness and applicability of *B. oleae* biocontrol strategies. Future research should be focused on discovering new virulent strains and the development of novel formulations for increased persistence, a longer shelf-life, and improved efficiency.

## Figures and Tables

**Table 1:** Isolates of EPF species that were tested in the present study.

Fungus Species	Isolate	Collection Site	Insect Bait
*Beauveria bassiana*	13.9	Elos Patras	*Sitophilus zeamais*
	13.20	Elos Patras	*Sitophilus zeamais*
	13.24	Dasylio Patras	*Tribolium confusum*
	13.27	Dasylio Patras	*Tribolium confusum*
	13.63	Dasylio Patras	*Sitophilus zeamais*
	13.65	Dasylio Patras	*Sitophilus zeamais*
	13.81	Elos Patras	*Tribolium confusum*
	13.96	Dasylio Patras	*Sitophilus zeamais*
	13.106	Elos Patras	*Tribolium confusum*
	13.108	Elos Patras	*Tribolium confusum*
	Achaia	Glafkos Achaia	*Plodia interpunctella*
	5339	Commercial	Commercial
*Beauveria varroae*	14	Elos Patras	*Sitophilus zeamais*
*Cordyceps blackwelliae*	17	Dasylio Patras	*Tribolium confusum*
*Metarhizium robertsii*	24	Elos Patras	*Tribolium confusum*
*Metarhizium anisopliae*	Crete	Messara Heraklion	*Sitophilus zeamais*

**Table 2:** ANOVA parameters of pupa hatch time, presence of mycelium on the pupae, adults’ survival time, presence of mycelium on the adults, and substrate for *B. oleae* pupae exposed to several isolates of *B. bassiana*, *B. varroae*, *M. anisopilae*, *M. robertsii*, and *C. blackwelliae.*

Factor	Df_(1,2)_	F	Sig.
**Pupa hatch time**			
Fungal isolates	16, 1320	2.110	0.014
Experiment duration	9, 1320	350.116	<0.001
Fungal isolates × experiment duration	144, 1320	3.113	<0.001
**Mycelium present on the pupae**			
Fungal isolates	16, 1320	3.233	<0.001
Substrate	1, 1320	5.116	<0.001
Fungal isolates × substrate	16, 1320	4.537	<0.001
**Adults’ survival time**			
Fungal isolates	16, 1320	0.650	0.934
Experiment duration	9, 1320	745.996	<0.001
Fungal isolates × experiment duration	144, 1320	4.893	<0.001
**Mycelium present on the adults**			
Fungal isolates	16, 1320	2.996	<0.001
Substrate	1, 1320	2.819	<0.001
Fungal isolates × substrate	16, 1320	1.835	<0.001
**Substrate**			
Pupa hatch time	16, 1320	1.656	0.024
Adult survival time	16, 1320	3.756	<0.001
Fungal isolates	16, 1320	4.110	<0.001
Pupa hatch time × adult survival time × fungal isolates	4.095, 1320	9.115	<0.001

**Table 3:** Pupa hatch time (days), mycelium on the pupae (%), adult survival time (days), and mycelium on dead adults (%) for *B. oleae*. Ten pupae were used per replication for twenty replications per isolate. Values with the same letter within a column are not significantly different (*p* < 0.05).

Treatment	Isolation Number	Substrate	Pupa Hatch Time (Days)	Mycelium on Pupae (%)	Adult Survival Time (Days)	Mycelium on Dead Adults (%)
Control	-	Soil	2.87 ± 0.15 a	0.0 ± 0.0 a	9.83 ± 083 a	0.0 ± 0.0 a
*Beauveria bassiana*	13.9	Soil	5.10 ± 0.50 b	28.57 ± 2.3 b	6.96 ± 0.42 b	43.48 ± 2.1 b
*Beauveria bassiana*	13.20	Soil	5.36 ± 0.56 b	71.43 ± 1.7 c	5.56 ± 0.42 b	86.96 ± 2.9 c
*Beauveria bassiana*	13.24	Soil	5.86 ± 0.58 b	54.55 ± 2.4 d	6.13 ± 0.46 b	84.21 ± 1.5 c
*Beauveria bassiana*	13.27	Soil	5.83 ± 0.54 b	63.64 ± 1.2 e	6.50 ± 0.50 b	73.68 ± 1.6 d
*Beauveria bassiana*	13.63	Soil	5.13 ± 0.54 b	87.50 ± 2.9 f	5.90 ± 0.46 b	86.36 ± 1.4 c
*Beauveria bassiana*	13.65	Soil	5.43 ± 0.46 b	83.33 ± 3.3 f	6.10 ± 0.36 b	75.00 ± 2.5 d
*Beauveria bassiana*	13.81	Soil	6.63 ± 0.18 c	100.00 ± 0.0 g	6.36 ± 0.40 b	81.82 ± 1.2 c
*Beauveria bassiana*	13.96	Soil	6.16 ± 0.08 b	90.00 ± 0.0 h	6.43 ± 0.47 b	80.00 ± 0.0 c
*Beauveria bassiana*	13.106	Soil	5.27 ± 0.59 b	58.33 ± 1.8 d	6.10 ± 0.49 b	72.22 ± 1.9 d
*Beauveria bassiana*	13.108	Soil	5.90 ± 0.18 b	85.71 ± 3.1 f	6.16 ± 0.45 b	91.30 ± 1.5 e
*Beauveria bassiana*	Achaia	Soil	5.10 ± 0.55 b	40.00 ± 0.0 i	6.16 ± 0.41 b	84.00 ± 2.7 d
*Beauveria bassiana*	5339	Soil	5.23 ± 0.41 b	55.56 ± 1.6 d	5.91 ± 0.33 b	95.24 ± 1.8 e
*Beauveria varroae*	14	Soil	5.80 ± 0.53 b	77.78 ± 3.1 c	6.43 ± 0.45 b	66.67 ± 1.0 f
*Cordyceps blackwelliae*	17	Soil	5.93 ± 0.05 b	88.89 ± 2.4 f	5.63 ± 0.43 b	100.00 ± 0.0 g
*Metarhizium anisopliae*	Crete	Soil	5.91 ± 0.24 b	62.50 ± 1.9 e	5.50 ± 0.42 b	100.00 ± 0.0 g
*Metarhizium robertsii*	24	Soil	6.33 ± 0.12 b	88.89 ± 2.7 f	5.33 ± 043 b	100.00 ± 0.0 g
Control	-	Non-soil	2.60 ± 0.10 a	0 ± 0 a	9.90 ± 0.72 a	0.0 ± 0.0 a
*Beauveria bassiana*	13.9	Non-soil	6.80 ± 0.17 b	100.00 ± 0.0 g	6.06 ± 0.22 b	0.0 ± 0.0 a
*Beauveria bassiana*	13.20	Non-soil	6.07 ± 0.29 c	81.82 ± 1.5 f	6.76 ± 0.46 b	15.79 ± 2.1 h
*Beauveria bassiana*	13.24	Non-soil	6.57 ± 0.10 b	100.00 ± 0.0 g	5.23 ± 0.45 bc	100.00 ± 0.0 g
*Beauveria bassiana*	13.27	Non-soil	6.23 ± 0.12 c	90.91 ± 2.4 h	5.43 ± 0.37 b	100.00 ± 0.0 g
*Beauveria bassiana*	13.63	Non-soil	5.53 ± 0.49 c	37.50 ± 2.7 i	6.90 ± 0.36 b	68.18 ± 1.1 f
*Beauveria bassiana*	13.65	Non-soil	5.97 ± 0.48 c	44.44 ± 1.1 i	6.86 ± 0.36 b	47.62 ± 1.3 b
*Beauveria bassiana*	13.81	Non-soil	5.90 ± 0.43 c	66.67 ± 2.1 e	7.50 ± 0.34 d	20.83 ± 2.1 i
*Beauveria bassiana*	13.96	Non-soil	5.97 ± 0.61 c	58.33 ± 1.5 d	7.26 ± 0.48 d	33.33 ± 2.6 j
*Beauveria bassiana*	13.106	Non-soil	6.67 ± 0.21 b	100.00 ± 0.0 g	7.13 ± 0.38 d	12.50 ± 1.7 h
*Beauveria bassiana*	13.108	Non-soil	6.03 ± 0.17 c	100.00 ± 0.0 g	7.93 ± 0.45 d	10.53 ± 1.0 h
*Beauveria varroae*	14	Non-soil	5.10 ± 0.51 c	57.14 ± 1.7 d	6.40 ± 0.41 b	0.00 ± 0.0 a
*Cordyceps blackwelliae*	17	Non-soil	5.37 ± 0.57 c	66.67 ± 2.1 c	6.36 ± 0.45 b	71.43 ± 2.2 d
*Metarhizium robertsii*	24	Non-soil	6.30 ± 0.17 c	100.00 ± 0.0 g	6.43 ± 0.45 b	0.00 ± 0.0 a
*Metarhizium anisopliae*	Crete	Non-soil	5.20 ± 0.43 c	16.67 ± 3.3 j	6.10 ± 0.45 b	0.00 ± 0.0 a
*Beauveria bassiana*	Achaia	Non-soil	5.70 ± 0.39 c	70.00 ± 0.0 c	6.63 ± 0.37 b	0.00 ± 0.0 a
*Beauveria bassiana*	5339	Non-soil	5.35 ± 0.33 c	40.00 ± 0.0 i	6.29 ± 0.70 b	57.89 ± 1.9 k

**Table 4:** Overall variables in the equation from Cox regression in terms of toxicity against pupae of *B. oleae*. All treatments tested had 1 df.

Treatments	B †	SE	Exp(B) ††	95.0% CI for Exp(B)	
				Lower	Upper
Control	−2.479	0.203	0.099	0.075	0.122
13.9	0.265	0.213	1.303	0.858	1.979
13.20	0.026	0.213	1.027	0.677	1.558
13.24	0.169	0.207	1.185	0.789	1.778
13.27	0.398	0.220	1.489	0.967	2.293
13.63	0.230	0.225	1.258	0.810	1.955
13.65	−0.074	0.221	0.928	0.602	1.432
13.81	0.220	0.213	1.245	0.820	1.891
13.96	−0.251	0.212	0.778	0.513	1.180
13.106	−0.048	0.209	0.953	0.633	1.434
13.108	0.380	0.224	1.462	0.943	2.266
Achaia	0.143	0.214	1.122	0.733	1.617
5339	0.465	0.216	0.691	0.442	1.130
14	0.512	0.215	1.668	1.095	2.542
17	0.320	0.209	1.377	0.909	2.085
Crete	−0.286	0.225	0.751	0.483	1.168
24	0.659	0.211	1.932	1.277	2.924

† B: B values are associated with an increased hazard and a decreased survival time; as the predictor increases, the hazard of the event increases, and the predicted survival duration decreases. Negative coefficients indicate decreased hazards and increased survival times. †† Exp(B): the ratio of the hazard rates.

**Table 5:** Overall variables in the equation from Cox regression for key factors in terms of toxicity against pupae of *B. oleae*. All treatments tested had 1 df.

Treatments	B †	SE	Exp(B) ††	95.0% CI for Exp(B)	
				Lower	Upper
Substrate	0.163	0.055	1.178	1.058	1.311
Pupa hatch time	0.337	0.156	1.401	1.032	1.903
Replication	−1.328	0.043	0.265	0.243	0.289
Adult survival time	0.550	0.212	1.901	1.674	2.145
Mycelium on pupae	0.110	0.202	1.780	1.573	1.986
Mycelium on dead adults	0.104	0.206	1.331	0.681	1.329

† B: B values are associated with increased hazards and decreased survival times; as the predictor increases, the hazard of the event increases, and the predicted survival duration decreases. Negative coefficients indicate decreased hazards and increased survival times. †† Exp(B): the ratio of the hazard rates.

## Data Availability

The data presented in this study are available on request from the corresponding author (S.M.).
